# Supplementary material for: E-cardiac patch to sense and repair infarcted myocardium
Source: Nat Commun. 2024 May 16;15:4133. doi: 10.1038/s41467-024-48468-x (PMC11099052; doi:10.1038/s41467-024-48468-x)
Supplement: Supplementary file 1 — Supplementary Information [file 41467_2024_48468_MOESM1_ESM.pdf]

Supplementary Materials for

E-cardiac patch to sense and repair infarcted myocardium

Renjie Qiu[1,2,3#], Xingying Zhang[4#], Chen Song[5#], Kaige Xu[4], Huijia Nong[1], Yi Li[6], Xianglong Xing[1], Kibret Mequanint[7], Qian Liu[8], Quan Yuan[9], Xiaomin Sun[2], Malcolm Xing[4]* and Leyu Wang[1,3]*

[1]Guangdong Provincial Key Laboratory of Construction and Detection in Tissue Engineering; Biomaterials Research Center, School of Biomedical Engineering, Southern Medical University, Guangzhou, Guangdong 510515, China.
[2]School of Traditional Chinese Medicine, Southern Medical University, Guangzhou, Guangdong 510515, China.
[3]Department of Anatomy, School of Basic Medical Sciences, Guangzhou Medical University, Guangzhou, Guangdong 511436, China.
[4]Department of Mechanical Engineering, University of Manitoba, Winnipeg, MB R3T 2N2, Canada.
[5]Central Laboratory, The Fifth Affiliated Hospital of Southern Medical University, Southern Medical University, Guangdong, Guangzhou 510900, China.
[6]Department of Biochemistry and Molecular Biology, School of Basic Medical Sciences; Guangdong Provincial Key Laboratory of Single Cell Technology and Application, Southern Medical University, Guangzhou, Guangdong Province, China.
[7]Department of Chemical and Biochemical Engineering, and School of Biomedical Engineering, The University of Western Ontario, London Ontario, N6A5B9, Canada.
[8]Department of Applied Computer Science, University of Winnipeg, Winnipeg, MB, R3B 2E9, Canada.
[9]State Key Laboratory of Oral Diseases & National Center for Stomatology & National Clinical Research Center for Oral Diseases, West China Hospital of Stomatology, Sichuan University, Chengdu 610041, Sichuan, China.

[#]These authors contribute equally to this work.
***Corresponding authors:**
wangleyu889@163.com; malcolm.xing@umanitoba.ca

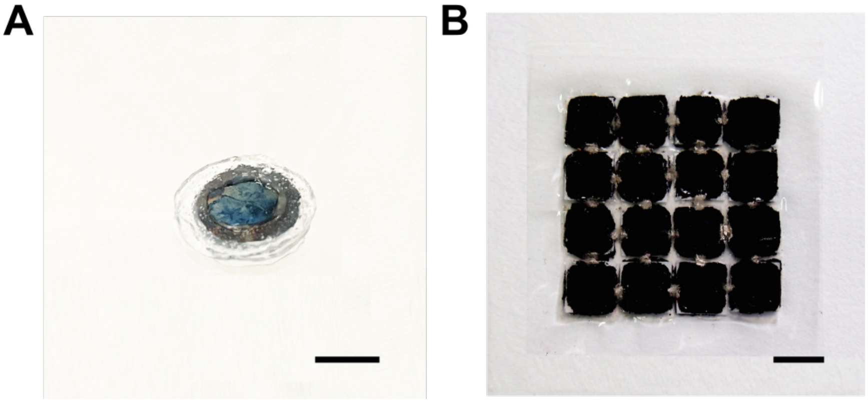

Supplementary Fig. 1 The physical pictures of the E-cardiac patch. (A) Round TRI-TENG exployed in cell and rat experiments. Scale bar: 0.5 cm. **(B)** TRI-TENG array used in large animal experiment. Scale bar: 1 cm.

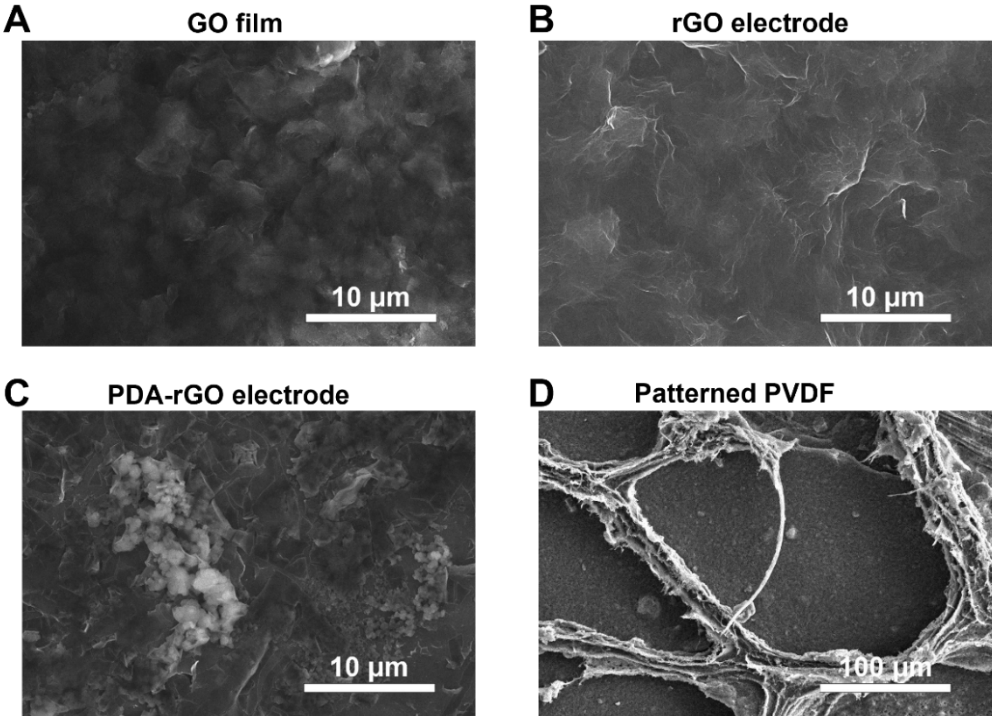

Supplementary Fig. 2 Scanning electron microscopy (SEM) graphs of GO film (A), rGO electrode (B), PDA-rGO electrode (C) and patterned PVDF with leaf vein structure (D).

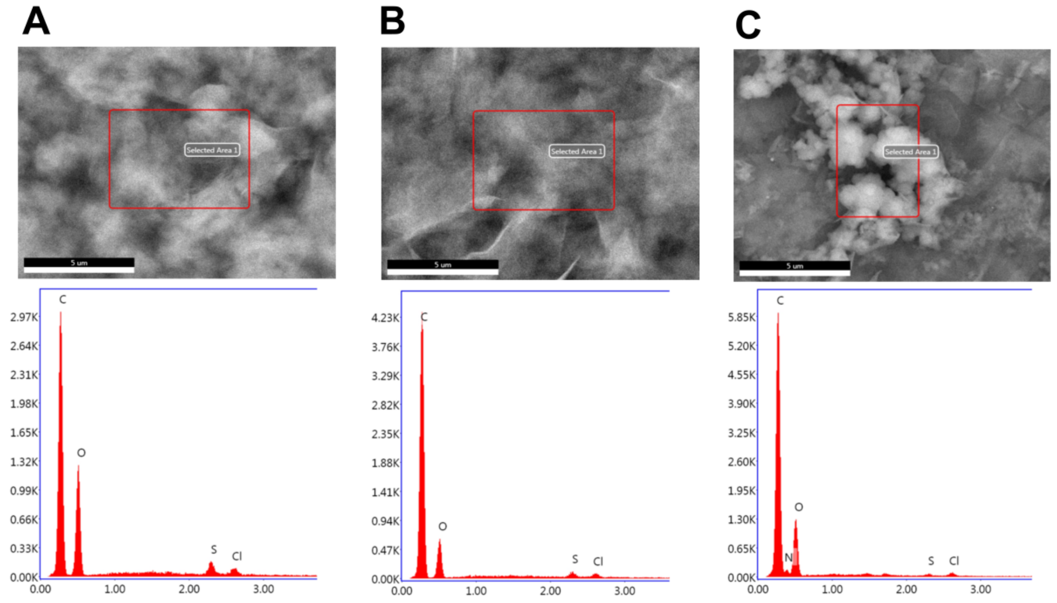

Element	Weight %	Atomic %	Error %
C K	59.95	67.4	6.43
O K	37.31	31.49	10.28
S K	1.62	0.68	9.56
Cl K	1.12	0.43	12.9

Element	Weight %	Atomic %	Error %
C K	74.38	80.07	5.2
O K	23.77	19.22	11.24
S K	0.97	0.39	12.4
Cl K	0.88	0.32	16.12

Element	Weight %	Atomic %	Error %
C K	59.6	65.77	4.99
N K	10.07	9.53	15.33
O K	29.37	24.33	10.59
S K	0.35	0.15	16.74
Cl K	0.61	0.23	14.86

Supplementary Fig. 3 Backscattered electron images, energy dispersive spectroscopy (EDS) analysis on selected areas, and tables of the elemental composition of GO film (A), rGO electrode (B), PDA-coated rGO electrode (C).

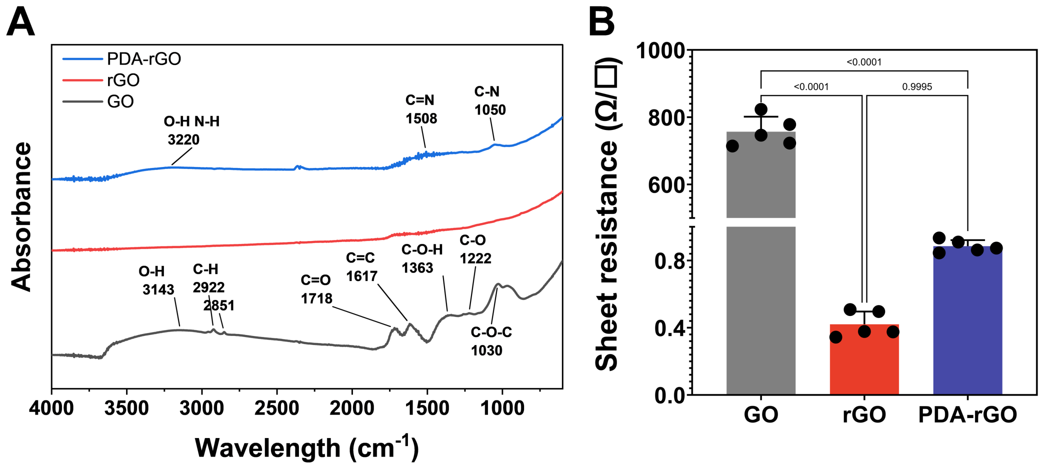

Supplementary Fig. 4. FTIR spectra and sheet resistance of different substrates. (A) FTIR spectra of GO film, rGO electrode and PDA-rGO electrode. **(B)** Sheet resistance of different substrates. n=5 independent samples. The data were presented as

mean ± SD. Statistical significance was calculated using two-side one-way ANOVA with Dunnett' s post-hoc test.

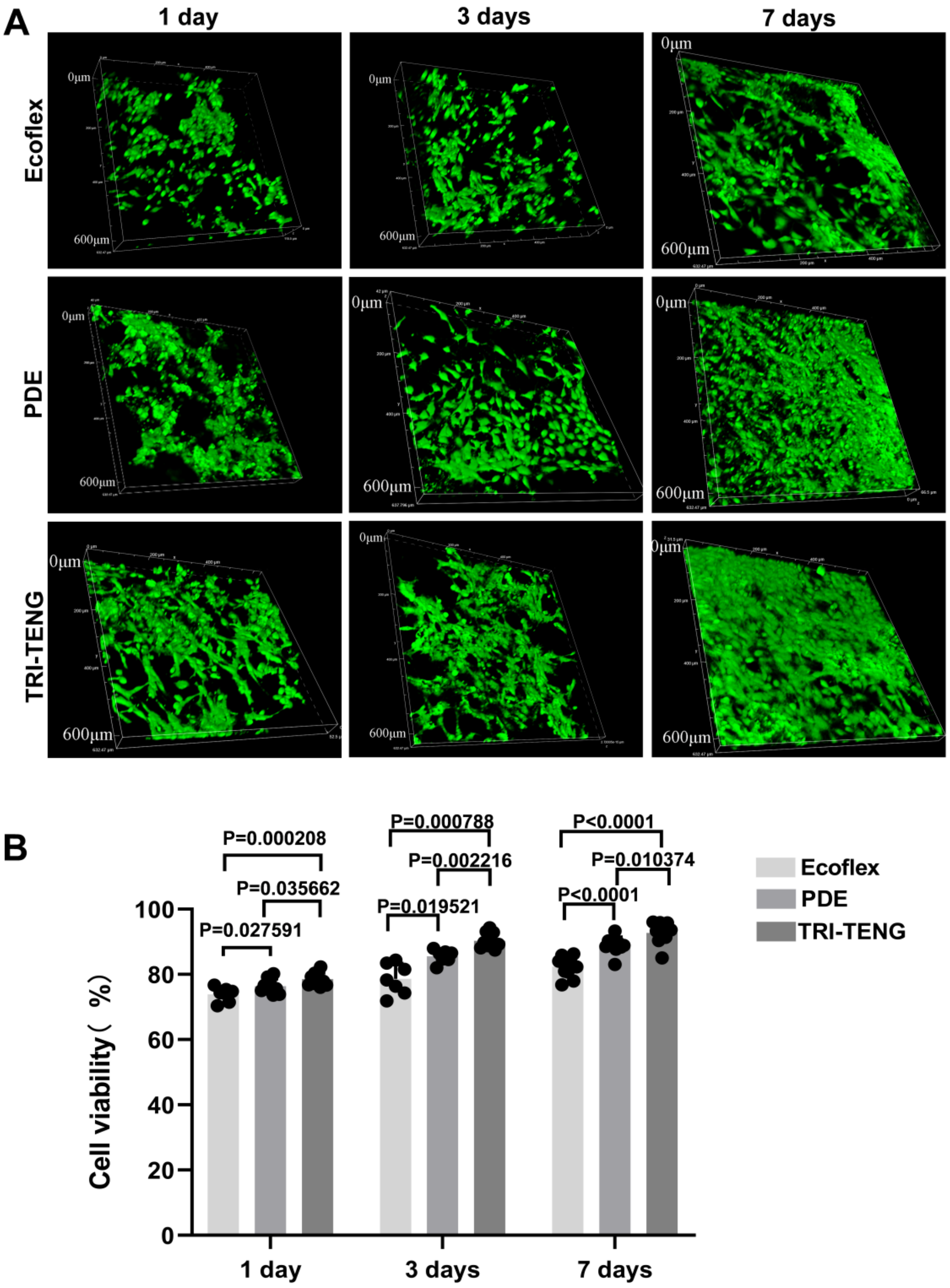

Supplementary Fig. 5 Biocompatibility evaluation for different scaffolds by live/dead staining assay. (A) Fluorescence 3D imaging of live CMs (green) and dead CMs (red) in different scaffolds at day 1, 3 ,7 of culture. **(B)** Quantitative cell viability of CMs in different groups based on fluorescence images. Each symbol denotes an field from 5 distinct samples (Ecoflex n=7, PDE n=10, and TRI-TENG n=10 on day 1.

Ecoflex n=7, PDE n=7, and TRI-TENG n=9 on day 3. n=10 in each group on day 7). The data were presented as mean ± SD. Statistical significance in samples on day 1 and 7 was calculated using two-side one-way ANOVA with LSD post-hoc test, and statistical significance in samples on day 3 was calculated using two-side one-way ANOVA with Dunnett' s post-hoc test.

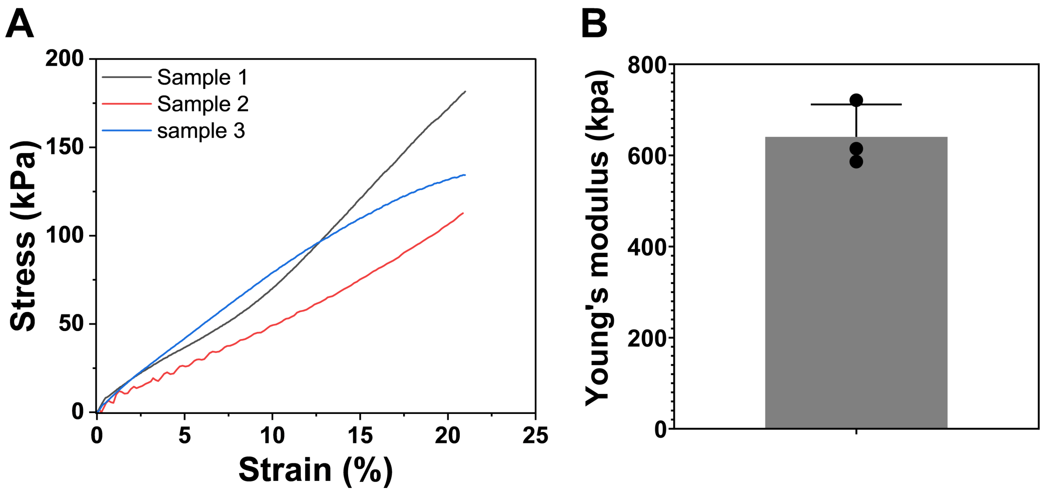

Supplementary Fig. 6 Mechanical properties of the TRI-TENG **(A)** Strain-stress curve and **(B)** Young's modulus. The data were presented as mean ± SD. n=3 independent samples.

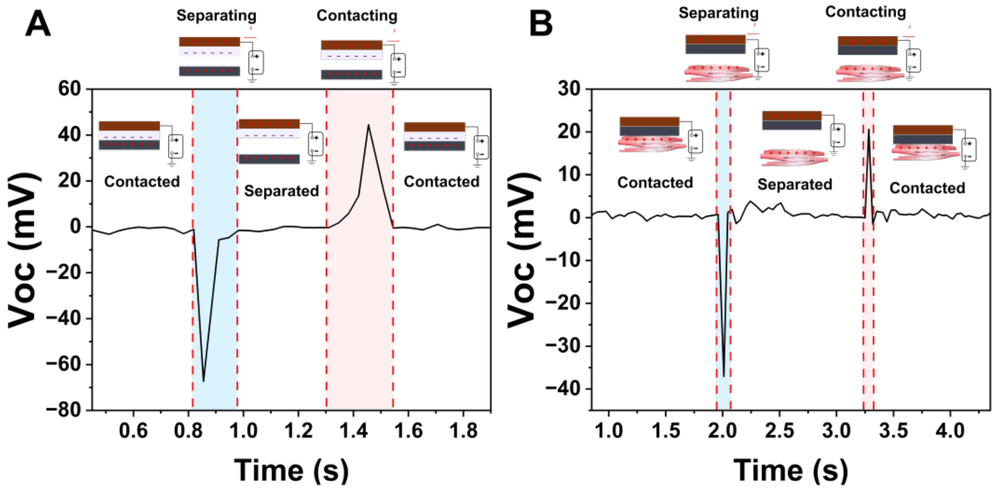

Supplementary Fig. 7 Voltage measured from a copper electrode (A) attached to the PVDF contacting with and separating from PDA-rGO and (B) attached to the PDA-rGO contacting with and separating from the myocardium.

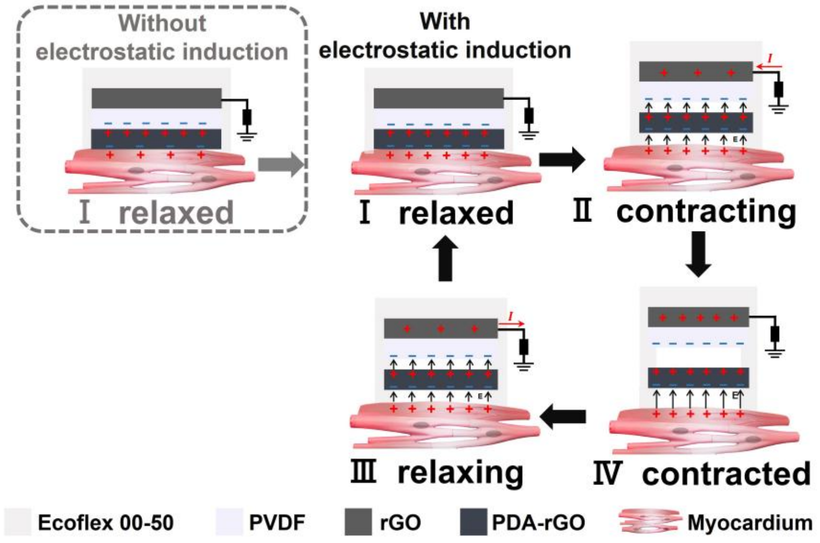

Supplementary Fig. 8 Working mechanism of the TRI-TENG as a sensor.

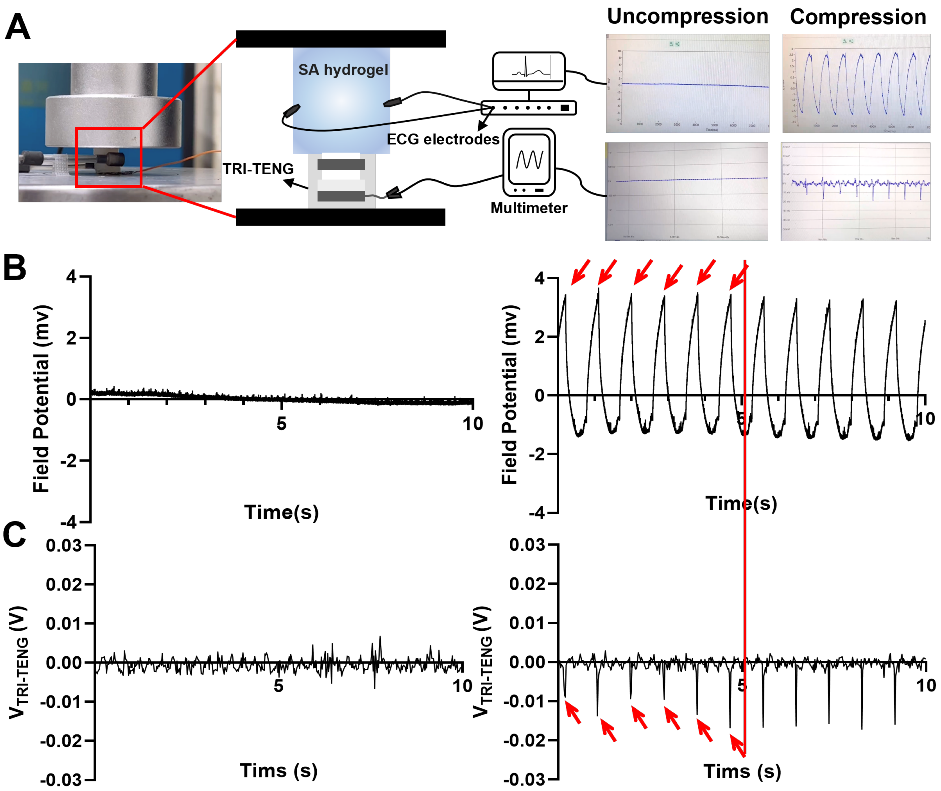

Supplementary Fig. 9 (A) Schematic illustration of the setup of the mode study. **(B)** Stimulation unit generated electrical field detected by the ECG electrode without the cyclic compression (left) and with the cyclic compression (right). **(C)** The voltage output of the sensing unit measured by the digital benchtop multimeter without the cyclic compression (left) and with the cyclic compression (right).

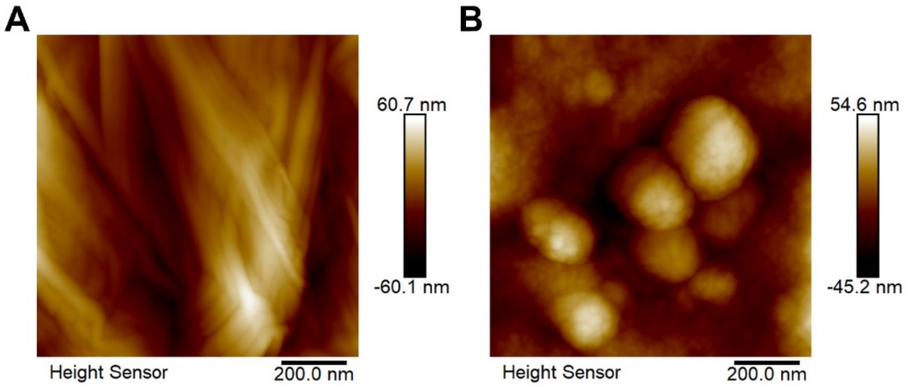

Supplementary Fig. 10 AFM images showing the surface of (A) rGO film and (B) PDA-rGO film.

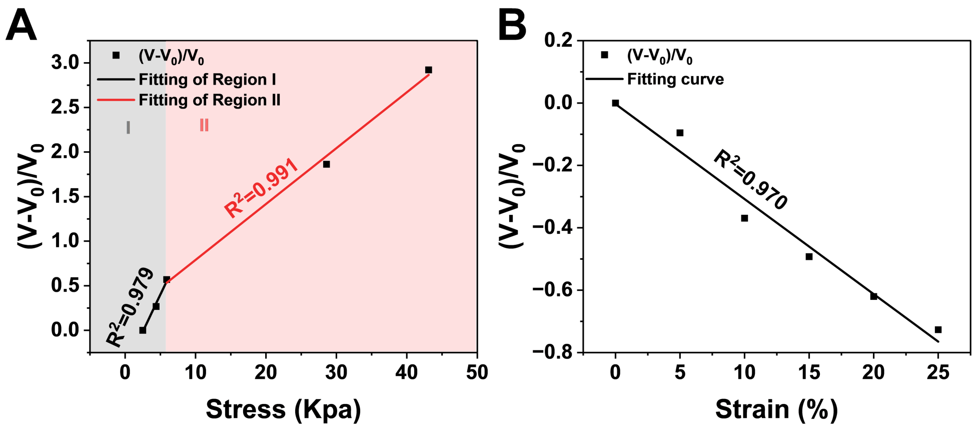

Supplementary Fig. 11 Linear fitting and the relationship between the relative change in voltage amplitude and different stimuli. (A) applied stress. **(B)** applied strain.

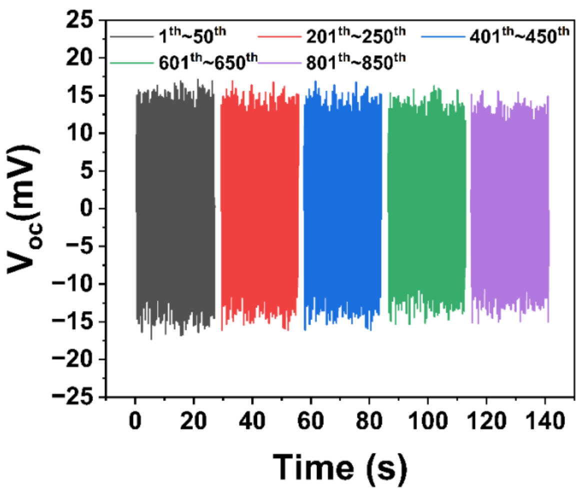

Supplementary Fig. 12 Voltage output of a TRI-TENG measured at different compression cycles.

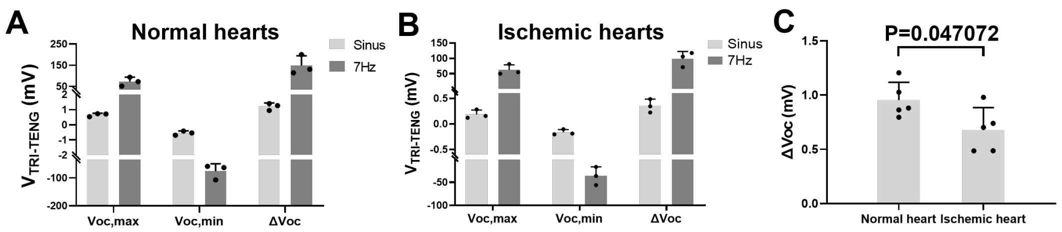

Supplementary Fig. 13 Statistics analysis of electrical output parameters from TRI-TENG. (A, B) The statistics analysis of $V_{OC,\,max}$, $V_{OC,\,min}$, and ΔV_{OC} obtained from TRI-TENG on Langendorff-perfused normal hearts (A) and ischemic rat hearts (B). n=3 independent rats. The signals were collected under sinus rhythm and 7 Hz stimulation pacing respectively. **(C)** Statistical comparisons of ΔVoc values by TRI-TENG in vivo rat hearts under the normal and ischemic states. n=5 independent rats. The data were presented as mean ± SD. Statistical significance was calculated using two-sided unpaired student’ s t-test.

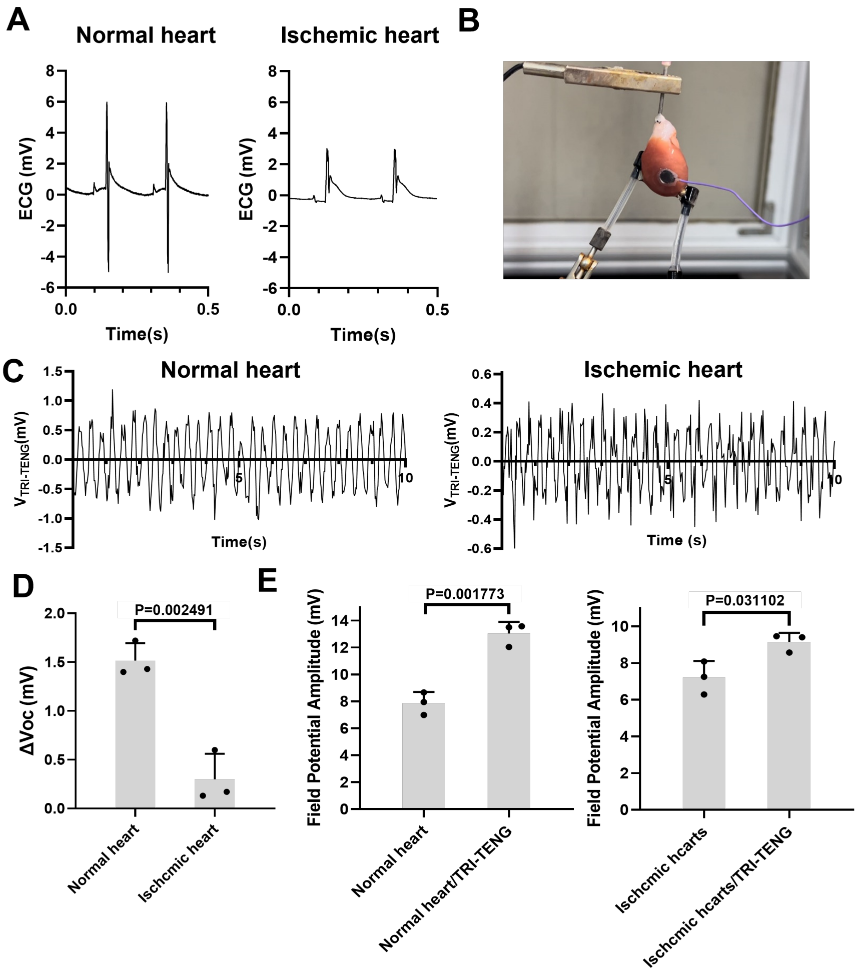

Supplementary Fig. 14 (A) Representative ECG traces of the normal heart and the ischemic heart without transplanted TRI-TENG. **(B)** Profile display of the ECG electrode placement for langendorff-perfused hearts, where TRI-TENG with wire was transplanted into the ischemic region. **(C)** Electrical signal outputs of TRI-TENG recorded from the TRI-TENG-transplanted Langendorff-perfused rat normal heart (left) and ischemic injured heart (right). **(D)** Statistics analysis of the ΔV_{OC} of normal and ischemic rat hearts. **(E)** Statistics analysis of the field potential amplitude obtained from Langendorff-perfused normal and ischemic rat hearts before and after TRI-TENG (without wire) transplantation. n=3 independent rats. The data were presented as mean ± SD. Statistical significance was calculated using two-sided unpaired student' s t-test.

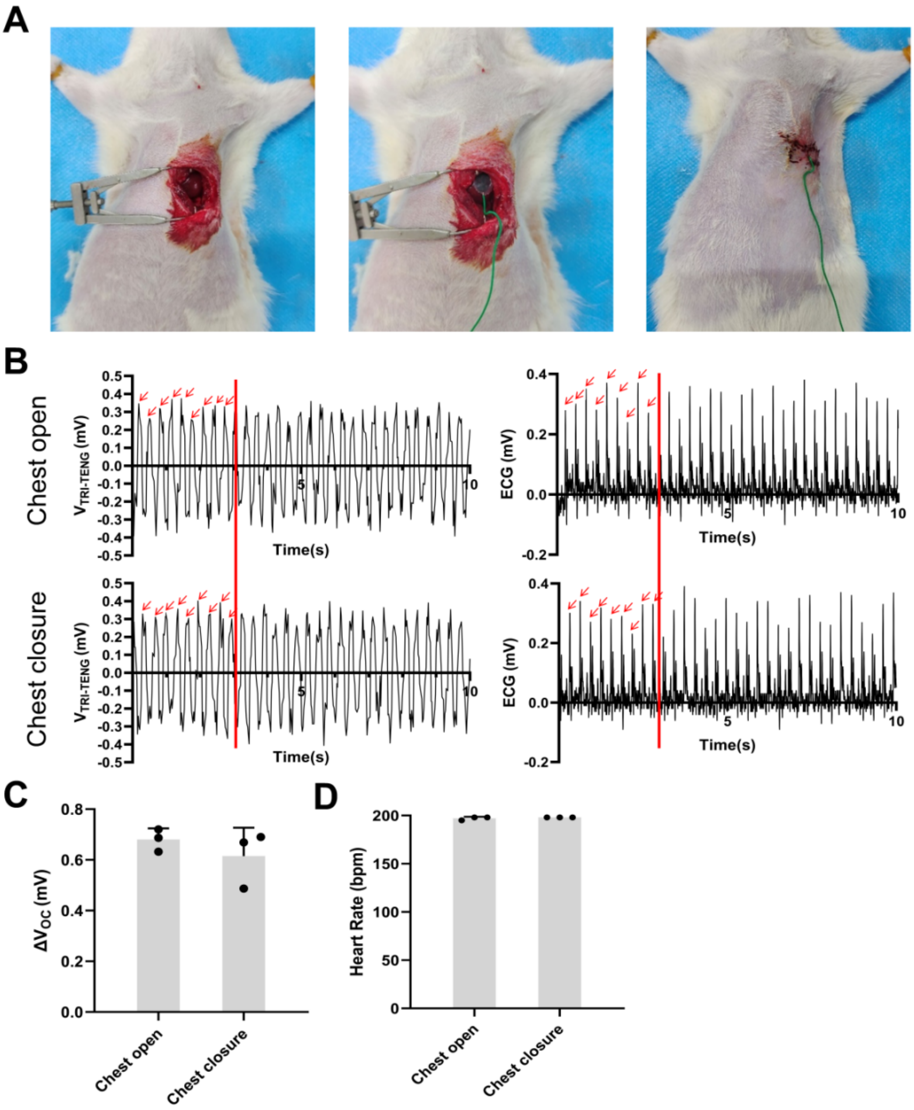

Supplementary Fig. 15 Electrical signal outputs of TRI-TENG and ECG signals recorded from rat hearts under both open-chest and closed-chest conditions. (A) Representative macroscopic images of TRI-TENG's electrical output assessment in rats under different conditions. **(B)** Open-circuit voltage (V_{OC}) values recorded from a rat heart transplanted with TRI-TENG under various conditions. The number of peaks in V_{OC} and in ECG marked by red arrows under open-chest conditions corresponds to those under closed-chest conditions. **(C, D)** Statistical analyses of the open-circuit voltage difference (ΔV_{OC}) (C) and heart rate (D) from the rat hearts. n=3 independent rats. The data were presented as mean ± SD.

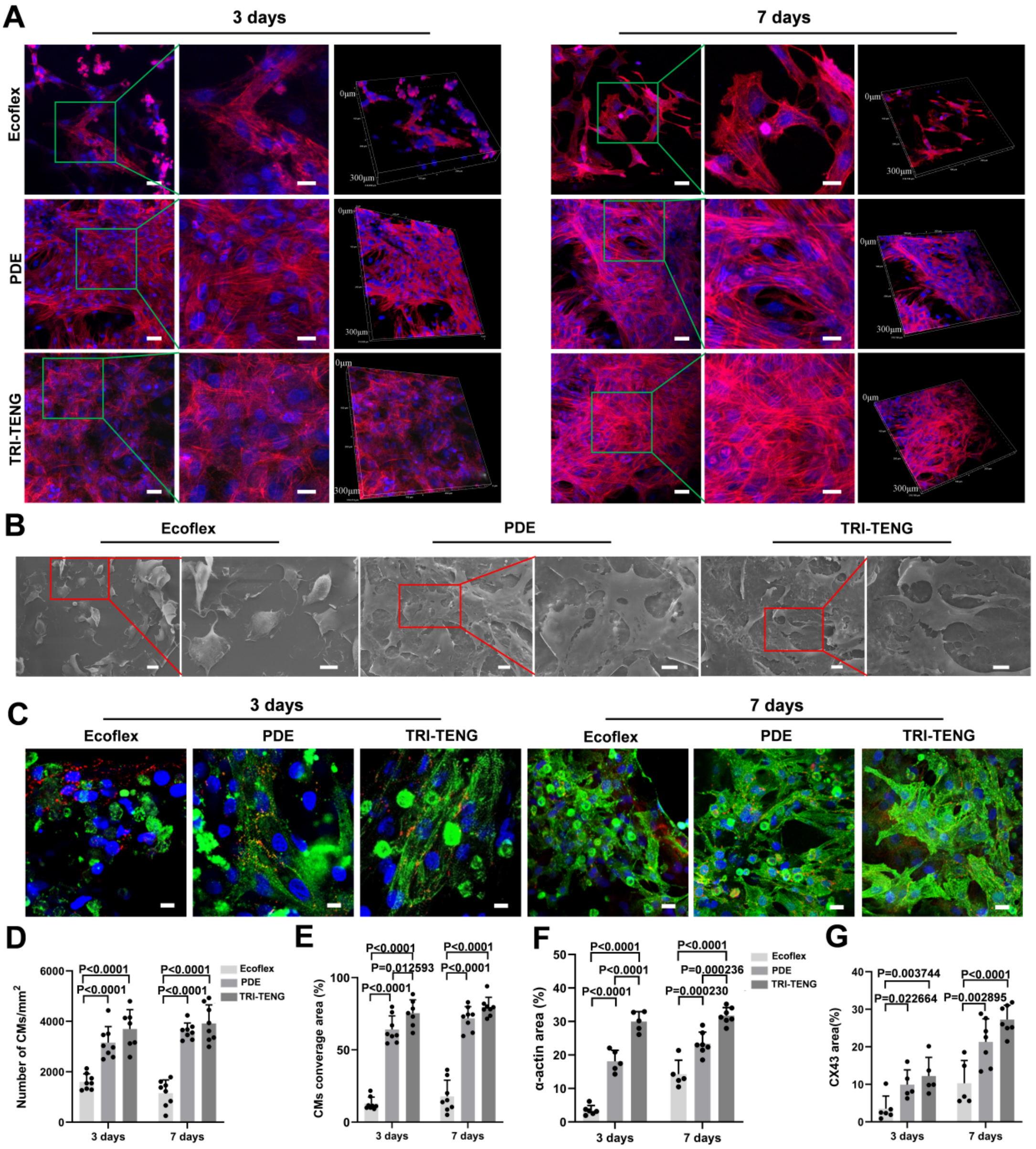

Supplementary Fig. 16 Morphology and specific protein expression of neonatal rat CMs on different scaffolds. (A) The 2D and 3D views of phalloidin (red) and DPAI (blue) staining in CMs in different CPs on days 3 and 7 of culture. Scale bars: 10 µm. **(B)** Scanning electron microscopy images of CMs on the Ecoflex, PDE and TRI-TENG. Scale bars: 20 µm. **(C)** Expressions of α-actinin (green) and CX43 (red) proteins in CMs in different CPs on days 3 and 7 of culture. Scale bars: 20 µm. **(D, E)** Quantification of CMs number (D) and CMs coverage area (E) based on the fluorescence images of phalloidin staining. Each symbol denotes a field from 5 distinct samples (Ecoflex n=8, PDE n=8, and TRI-TENG n=7 on day 3. n=8 in each group on day 7). **(F, G)** Quantitative analyses of α-actinin (F) and CX43 (G) fluorescent intensity.

Each symbol denotes a field from 5 distinct samples (Ecoflex n=6, PDE n=5, and TRI-TENG n=5 on day 3. Ecoflex n=5, PDE n=7, and TRI-TENG n=7 on day 7). The data were presented as mean ± SD. Statistical significance in samples of (D) on day 7 was calculated using two-side one-way ANOVA with Dunnett's post-hoc test, and statistical significance in the other samples was calculated using two-side one-way ANOVA with LSD post-hoc test.

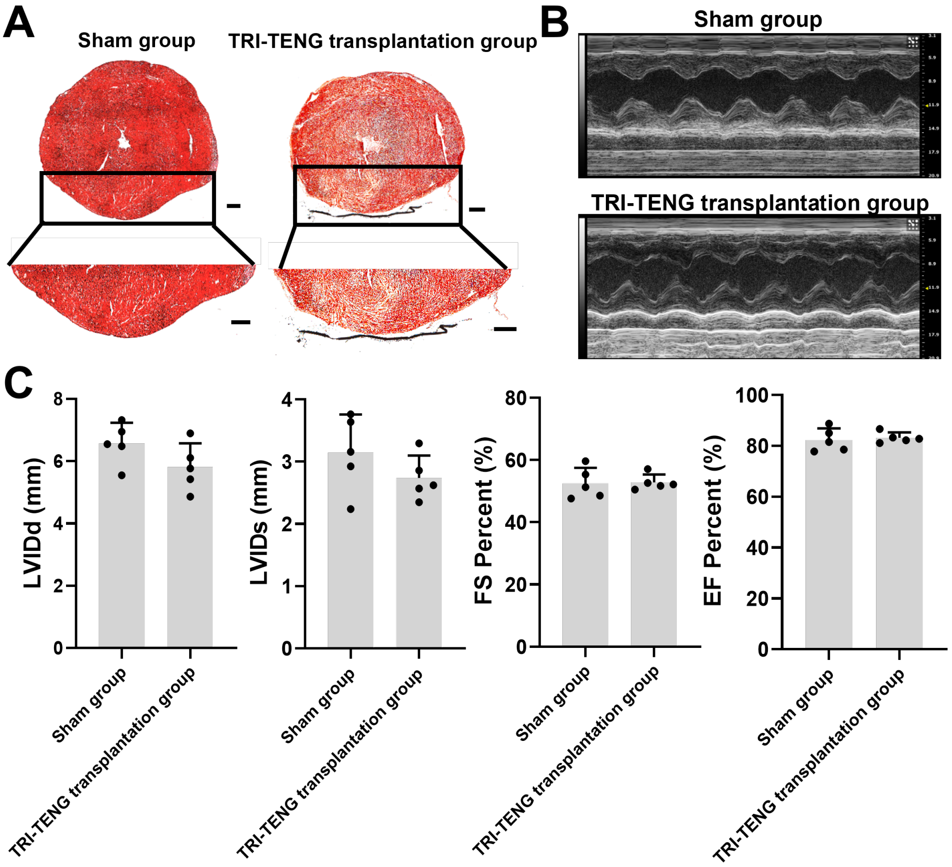

Supplementary Fig. 17 Histological examination and assessment of cardiac function in rat hearts from the sham group and the TRI-TENG transplanted group at week 4 post-operation. (A) Masson's Trichrome staining for cardiac sections in two groups. Red: myocardium. Scale bars: 1 mm. **(B)** Echocardiograms of left ventricular (LV) contraction in two groups. **(C)** Values of Left ventricle internal diameter in diastole (LVIDd), Left ventricle internal diameter in systole (LVIDs), Fraction Shorting (FS) and Ejection Fraction (EF) determined by echocardiography in two groups at week 4 post-operation. n=5 independent rats. The data were presented as

mean ± SD. Statistical significance was calculated using two-sided unpaired student’ s t-test.

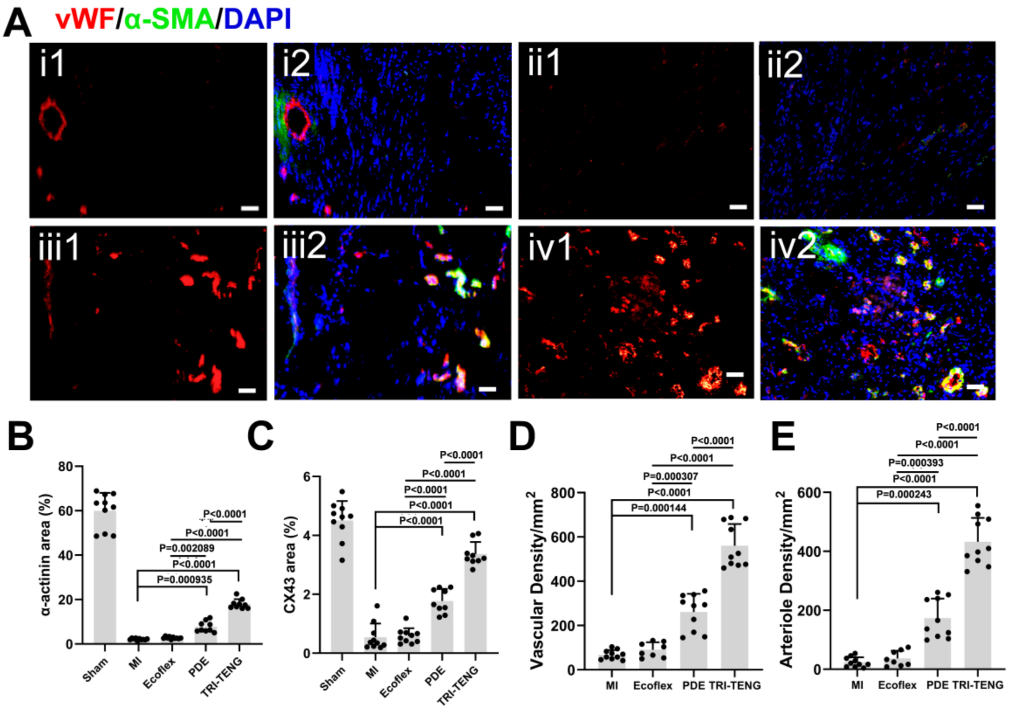

Supplementary Fig. 18 The histological analysis for the cardiac sections in different groups in rat MI models at week 4 after transplantation. (A) vWF positive proteins (red) and α-SMA positive proteins (green) in the infarct regions in various groups. Scale bars: 10 μm. **i:** MI group, **ii:** Ecoflex group, **iii:** PDE group, **iv:** TRI-TENG group. **(B-E)** Statistical comparisons of the percentages of α-actinin area (B), CX43 area (C), vascular density (D), and arteriole density (E) in the infarct regions in different groups based on the immunostaining images. Vascular density was determined by counting vWF^{+} vessels, and arteriole density was determined by $vWF^{+}/\alpha\text{-}SMA^{+}$ vessels. Each symbol denotes an independent rat (Sham n=10, MI n=10, Ecoflex n=10, PDE n=9, and TRI-TENG n=9 in the B and C. MI n=10, Ecoflex n=8, PDE n=10, and TRI-TENG n=10 in the D and E). The data were presented as mean ± SD. Statistical significance in (B), (D) and (E) was calculated using two-side one-way ANOVA with Dunnett’ s post-hoc test, and statistical significance in (C) was calculated using two-side one-way ANOVA with LSD post-hoc test.

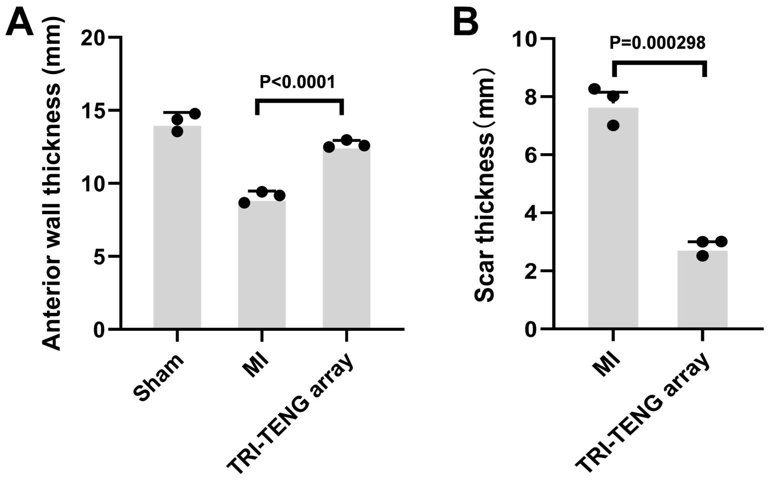

Supplementary Fig. 19 Morphometry results showed the anterior wall thickness (A) and the scar thickness (B) of the LV in different groups in porcine MI models. n=3 independent minipigs. The data were presented as mean ± SD. Statistical significance in (A) was calculated using two-side one-way ANOVA with LSD post-hoc test, and statistical significance in (B) was calculated using two-sided unpaired student' s t-test.

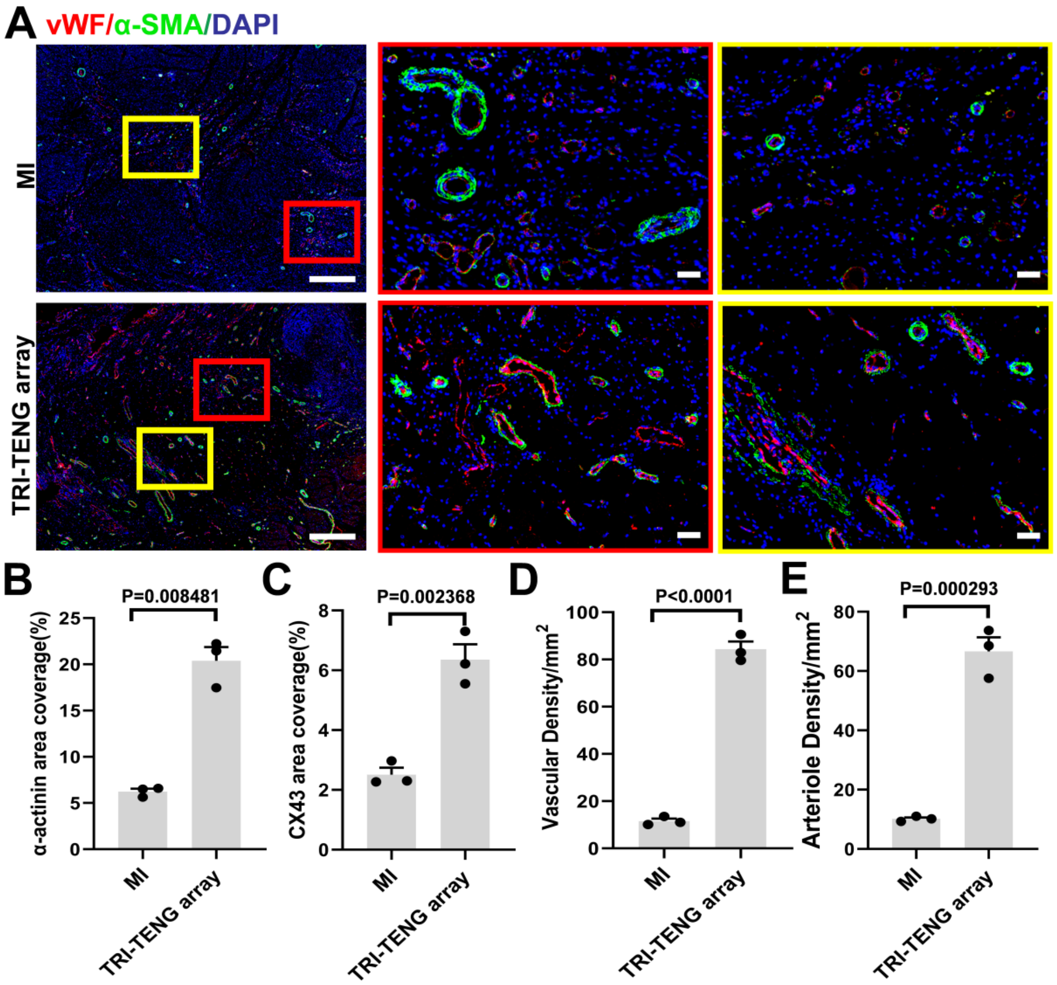

Supplementary Fig. 20 Revascularization evaluation for the porcine cardiac sections in infarcted regions in different groups. (A) Double immunofluorescent staining of vWF/α-SMA in porcine cardiac sections in different groups. Scale bars, 500 µm (left) and 50 µm (right). **(B-E)** α-actinin area coverage (B), CX43 area coverage (C), vascular density (D), and arteriole density (E) in cardiac sections calculated from the immunostaining images. n=3 independent minipigs. The data were presented as mean ± SD. Statistical significance was calculated using two-sided unpaired student' s t-test.

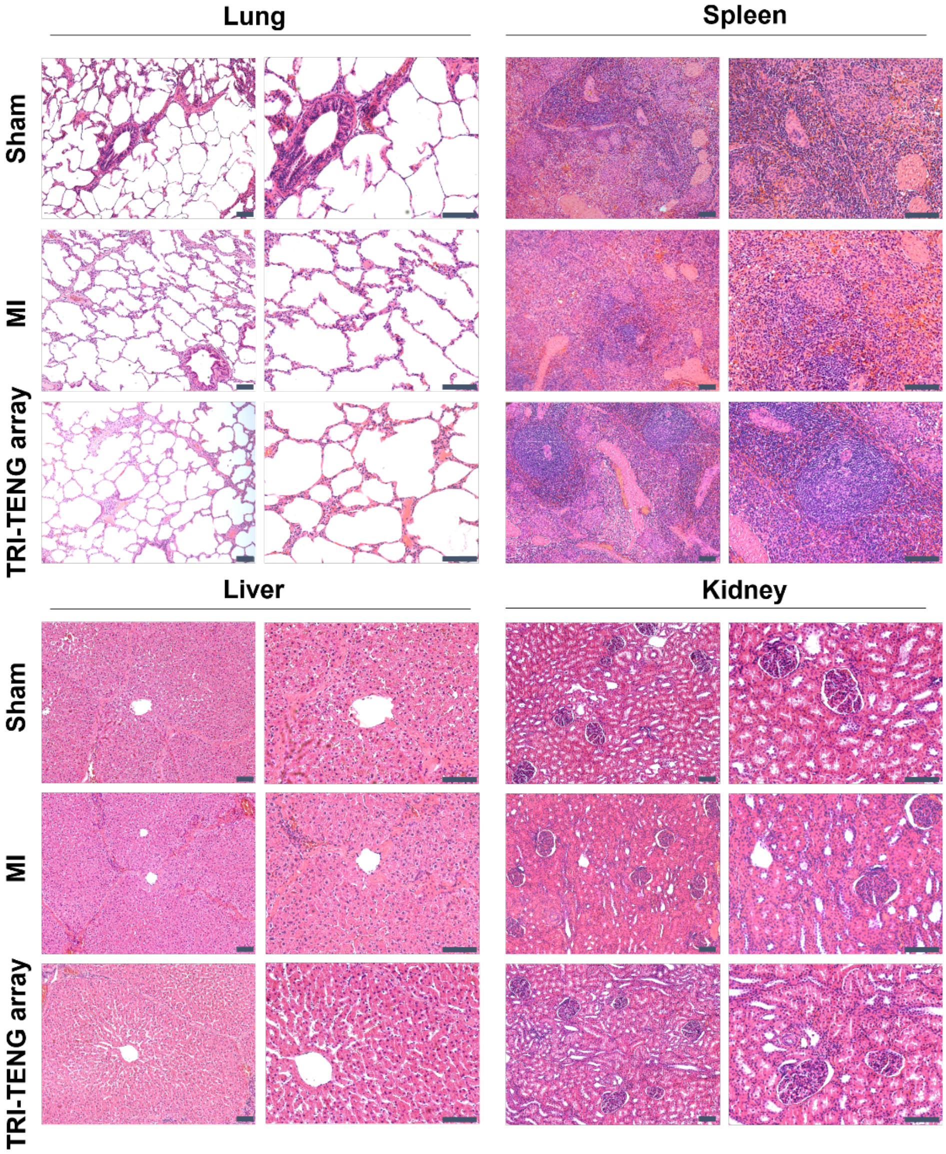

Supplementary Fig. 21 HE staining for the lungs, spleens, livers and kidneys of minipigs in different groups at week 4 post-transplantation.

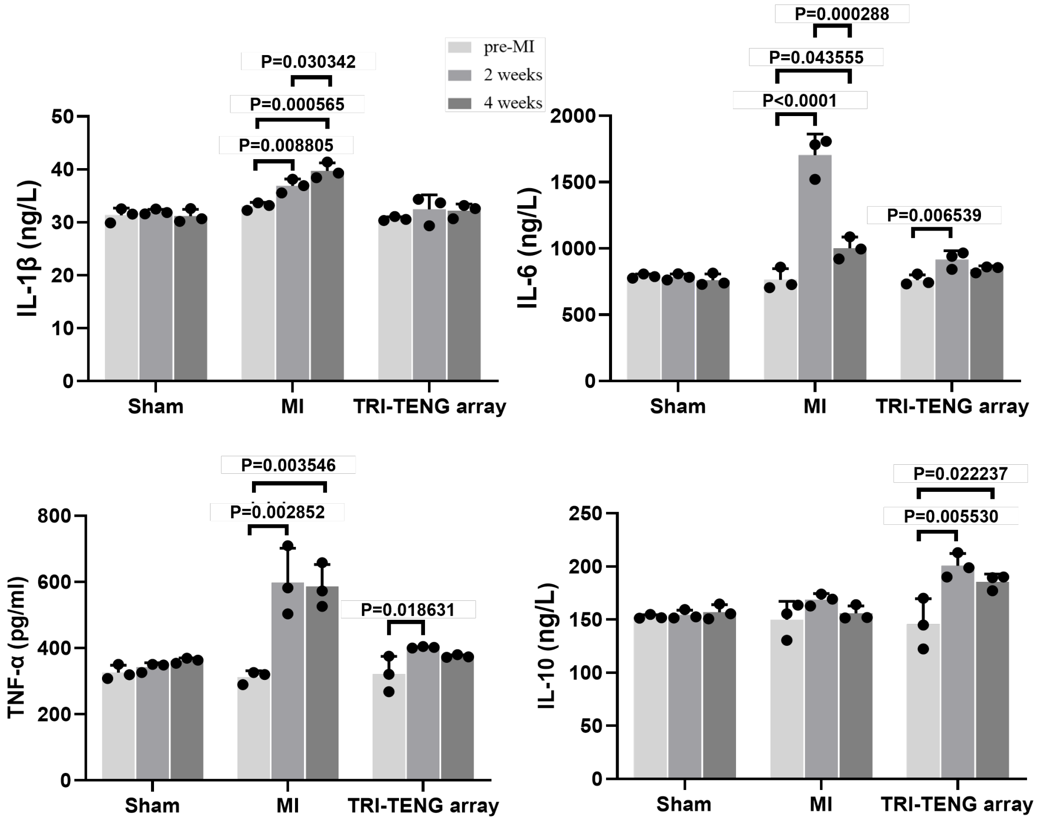

Supplementary Fig. 22 Elisa detection for inflammatory cytokines from blood samples in minipigs in different groups at the predetermined times. n=3 independent minipigs**.** The data were presented as mean ± SD. Statistical significance was calculated using two-side one-way ANOVA with LSD post-hoc test.

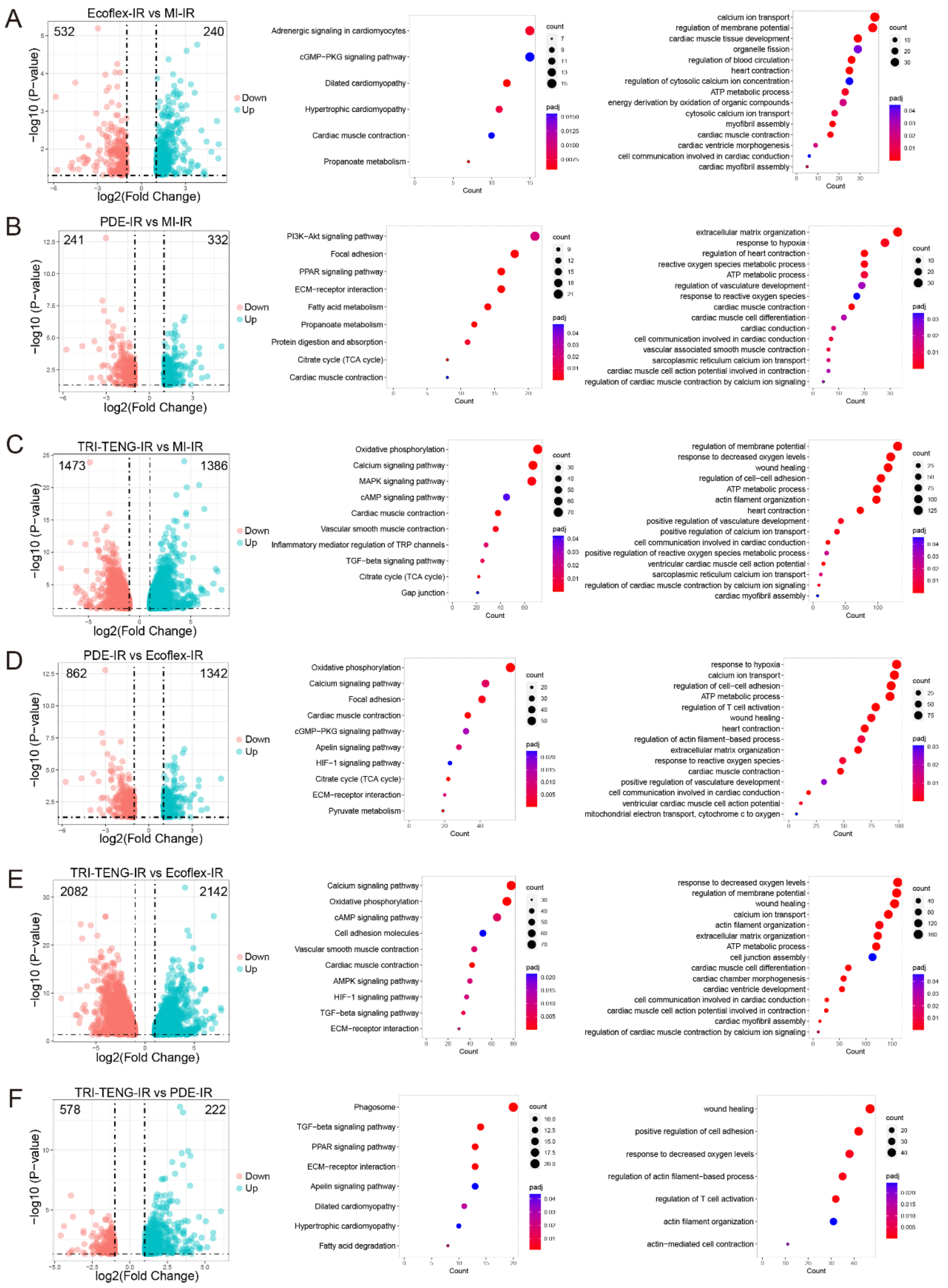

Supplementary Fig. 23 The functional analysis of differentially expressed genes (DEGs) in infarct region (IR) of different groups at week 4 after transplantation. Volcano plot displaying the DEGs in each pairwise comparison among all groups (left in A-F). Red dots represent down-regulated expressed genes and blue dots represent up-regulated expressed genes. KEGG pathway enrichment scatter plot in each pairwise

comparison among all groups (middle in A-F). The x-axis indicates the number of enrichment genes, and the y-axis related KEGG pathway. Gene Ontology (GO) enrichment of the biological process (BP) category in each pairwise comparison among all groups (right in A-F). The x-axis indicates the number of enrichment genes in GO BP term, and the y-axis is related to GO BP terms.

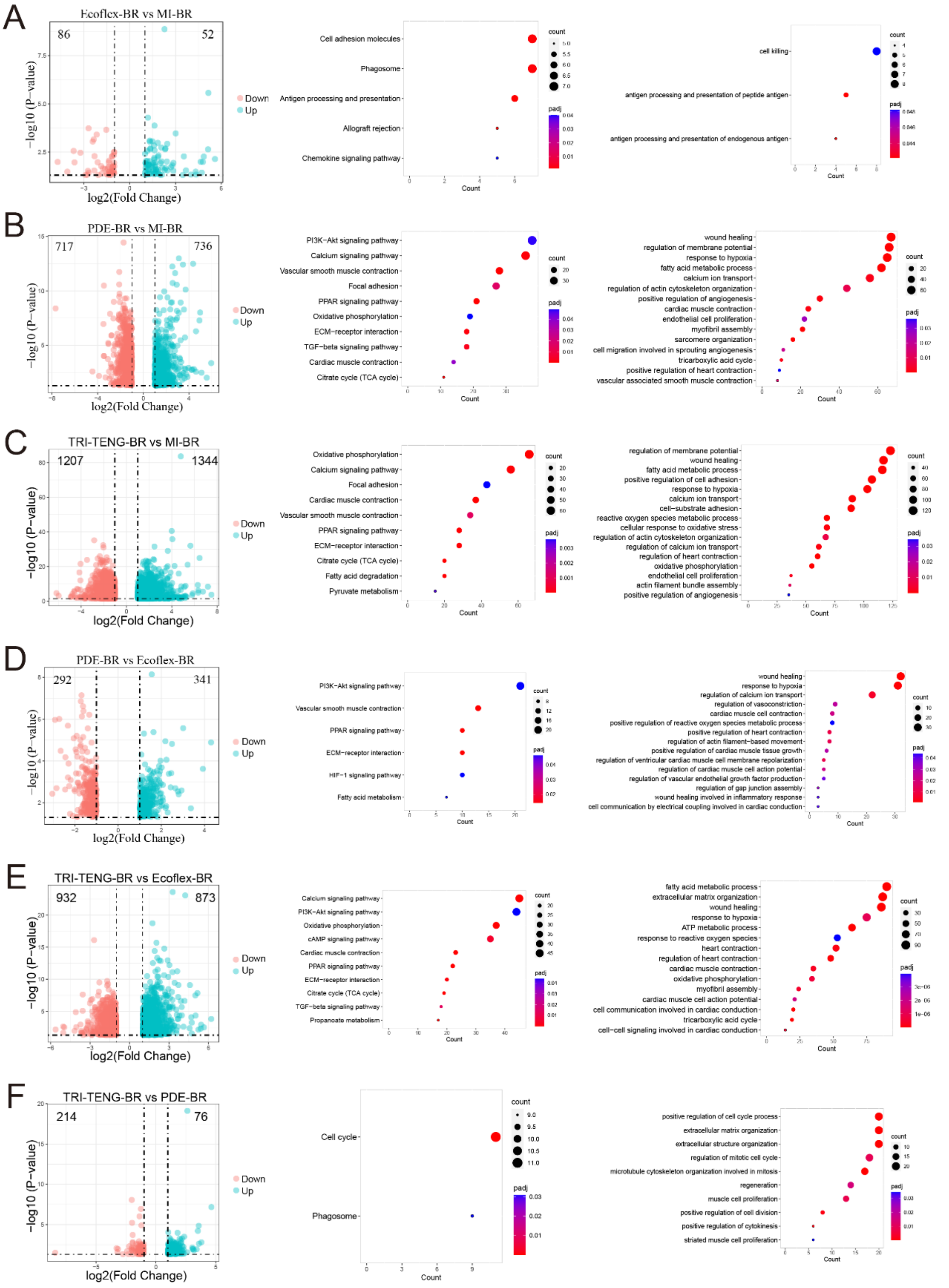

Supplementary Fig. 24 The functional analysis of DEGs in border region (BR) of different groups at week 4 after transplantation. Volcano plot displaying the DEGs in each pairwise comparison among all groups (left in A-F). Red dots represent down-regulated expressed genes and blue dots represent up-regulated expressed genes.

KEGG pathway enrichment scatter plot in each pairwise comparison among all groups (middle in A-F). The x-axis indicates the number of enrichment genes, and the y-axis related KEGG pathway. Gene Ontology (GO) enrichment of the biological process (BP) category in each pairwise comparison among all groups (right in A-F). The x-axis indicates the number of enrichment genes in GO BP term, and the y-axis is related to GO BP terms.

Supplementary Table 1. The cell viability of different implantable self-powered materials.

**Implantable self-powered materials**	**Cell viability**	**Application**	**Ref.**
TRI-TENG	Over 80%	MI repair	Our work
Injectable TENG (I-TENG)	Over 90%	Wound healing	1
Composed of a self-powered TENG and an implantable nitric oxide (NO) releasing device	Over 90%	Intracranial neuroglioma therapy	2
Bioabsorbable natural-materials-based TENG (BN-TENG)	Over 95%	Improving dysfunctional cardiomyocyte contraction *in vitro*	3
Composed of TENG and interdigitated electrode	Over 80%	Promoting the maturation of cardiomyocytes *in vitro*	4
Piezoelectric acrylate epoxidized soybean oil (AESO) scaffolds doped with piezoelectric Ag-TMSPM-pBT (ATP) nanoparticles (AESO-ATP scaffolds)	Over 80%	Bone regeneration	5
Composite scaffold consisting of $BaTiO_3$ coated on porous Ti6Al4V	Over 80%	Repairing bone defects	6
$CaCO_3$-mineralized piezoelectric biodegradable scaffolds based on two polymers: poly[(R)3-hydroxybutyrate] (PHB) and poly[3-hydroxybutyrate-co-3-hydroxyvalerate] (PHBV)	Over 70%	Stimulating the growth of bone tissue	7
Injectable T-BTO-nanoparticles-embedded thermosensitive hydrogel	Over 80%	Tumor eradication	8
Dynamically evolving nanocomposites	Over 80%	Wound healing	9

Supplementary Table 2. The data of pacing thresholds of Langendorff-perfused rat hearts in different groups under normal conditions.

Sample \ Threshold Voltage(V) \ Group	Control	Ecoflex	PDE	TRI-TENG
1	2.5V	2.5V	2.5V	1.5V
2	2.5V	2.5V	2.5V	1.5V
3	2.5V	2.5V	2.5V	2V
4	2V	2V	2V	1.5V
5	2.5V	2.5V	2.5V	1.5V
6	2V	2V	2V	1.5V

Supplementary Table 3. The data of pacing thresholds of Langendorff-perfused rat hearts in different groups under ischemic conditions.

Sample \ Threshold Voltage(V) \ Group	MI	Ecoflex	PDE	TRI-TENG
1	2V	2V	2V	1.5V
2	2.5V	2.5V	2.5V	1V
3	2.5V	2.5V	2.5V	1.5V
4	2V	2V	2V	1V
5	2.5V	2.5V	2.5V	1.5V
6	2.5V	2.5V	2.5V	2V

References:

1. Xiao X, *et al.* Ultrasound-driven injectable and fully biodegradable triboelectric nanogenerators. *Small Methods* **7**, 2201350 (2023).
2. Yao S, *et al.* Self-Powered, Implantable, and Wirelessly Controlled NO Generation System for Intracranial Neuroglioma Therapy. *Adv Mater* **34**, 2205881 (2022).
3. Jiang W, *et al.* Fully bioabsorbable natural-materials-based triboelectric nanogenerators. *Adv Mater* **30**, 1801895 (2018).
4. Zhao L, *et al.* Promoting maturation and contractile function of neonatal rat cardiomyocytes by self-powered implantable triboelectric nanogenerator. *Nano Energy* **103**, 107798 (2022).
5. Li G, Li Z, Min Y, Chen S, Han R, Zhao Z. 3D-Printed Piezoelectric Scaffolds with Shape Memory Polymer for Bone Regeneration. *Small*, 2302927 (2023).
6. Liu W, *et al.* Biological effects of a three-dimensionally printed Ti6Al4V scaffold coated with piezoelectric BaTiO3 nanoparticles on bone formation. *ACS Appl Mater Interfaces* **12**, 51885-51903 (2020).
7. Chernozem R, *et al.* Piezoelectric 3-D fibrous poly (3-hydroxybutyrate)-based scaffolds ultrasound-mineralized with calcium carbonate for bone tissue engineering: inorganic phase formation, osteoblast cell adhesion, and proliferation. *ACS Appl Mater Interfaces* **11**, 19522-19533 (2019).
8. Zhu P, Chen Y, Shi J. Piezocatalytic tumor therapy by ultrasound-triggered and BaTiO3-mediated piezoelectricity. *Adv Mater* **32**, 2001976 (2020).
9. Zhu Z, *et al.* Dynamically evolving piezoelectric nanocomposites for antibacterial and repair-promoting applications in infected wound healing. *Acta Biomaterialia* **157**, 566-577 (2023).
