# Supplementary material for: E-cardiac patch to sense and repair infarcted myocardium
Source: Nat Commun. 2024 May 16;15:4133. doi: 10.1038/s41467-024-48468-x (PMC11099052; doi:10.1038/s41467-024-48468-x)
Supplement: Supplementary file 3 — Description of Additional Supplementary Files [file 41467_2024_48468_MOESM3_ESM.pdf]

<u>Description of Additional Supplementary Files</u>

Supplementary Movie 1 to Supplementary Movie 7

Supplementary Movie 1 ： A setup of the model study as well as the ECG of the hydrogel and the voltage output of the TRI-TENG under cyclic compression.

Supplementary Movie 2 ： TRI-TENG small strain monitoring.

Supplementary Movie 3 ： TRI-TENG large strain monitoring.

Supplementary Movie 4 ： TRI-TENG activity monitoring.

Supplementary Movie 5 ： TRI-TENG serves as a self-sustaining wireless sensor for monitoring rat electrocardiosignal during both normal and ischemic states.

Supplementary Movie 6 ： The echocardiography video of rats’ left ventricle contraction in different groups at week 4 after transplantation.

Supplementary Movie 7 ： The echocardiography video of minipigs’ left ventricle contraction in different groups at week 4 after transplantation.

Supplementary Datasets

Supplementary Data 1. The application of Triboelectric Nanogenerator (TENG) in cardiovascular healthcare.

Supplementary Data 2. Progress in the treatment of myocardial infarction (MI) in minipigs over the past five years.

Supplementary Data 3: Whole blood panel analysis of minipigs at pre-MI, 2 weeks and 4 weeks after TRI-TENG array transplantation.

Supplementary Data 4: The porcine kidney and liver function at pre-MI, 2 weeks and 4 weeks after TRI-TENG array transplantation.
