# Supplementary material for: E-cardiac patch to sense and repair infarcted myocardium
Source: Nat Commun. 2024 May 16;15:4133. doi: 10.1038/s41467-024-48468-x (PMC11099052; doi:10.1038/s41467-024-48468-x)
Supplement: Supplementary file 17 — Response to Editorial Requests [file 41467_2024_48468_MOESM17_ESM.docx]

*Please remove legends of Supplementary Data 1 and 2 from the Supplementary Information file, They can be given in author Checklist.
Response: Those legends were removed and the author checklist was updated.

*It seems Table 4 is given outside the Supplementary Information file, please note Supplementary Tables larger than A4 size should be given as Supplementary Datasets as you have given for Data 1 and 2. Please rename it accordingly in the Supplementary Information and provide the citation in article file.
Response: Data 3 (Table 4) has been added and meanwhile Table 5 ( larger than A4 size) was also changed to Data 4.
+++++++++++++++
